# Men Who Have Sex with Both Men and Women in West Africa: Factors Associated with a High Behavioral Risk of Acquiring HIV from Male Partners and Transmission to Women (CohMSM ANRS 12324—Expertise France)

**DOI:** 10.1007/s10508-023-02715-2

**Published:** 2023-11-16

**Authors:** Marion Fiorentino, Bakary Coulibaly, Clotilde Couderc, Bintou Dembélé Keita, Camille Anoma, Elias Dah, Ephrem Mensah, Thomas Niamkey Aka, Juste Rodrigue Touré, Drissa Camara, Anouwarsadat Rodolphe Kokouba, Gwenaëlle Maradan, Marion Mora, Michel Bourrelly, Lucas Riegel, Daniela Rojas-Castro, Bruno Spire, Christian Laurent, Luis Sagaon-Teyssier, The CohMSM Study Group, Véronique Boyer, Eric Delaporte, Bruno Granouillac, Suzanne Izard, Laura March, Martine Peeters, Laetitia Serrano, Cyril Berenger, Pierre-Julien Coulaud, Bakri M’madi Mrenda, Enzo Parisi, Adeline Bernier, Emmanuel Trenado, Oumar Cisse, Alou Coulibaly, Bintou Dembele Keita, Fodié Diallo, Mahamadou Diarra, Mady Gadjigo, Abdoul Aziz Keita, Kader Maiga, Fodé Traore, Niamkey Thomas Aka, Malan Jean-Baptiste Kouame, Frédéric Dibi N’guessan, Xavier Anglaret, Stéphane-Alain Babo Yoro, Jean-Marie Masumbuko, Maxime Oga, Christian Coulibaly, Ter Tiero Elias Dah, Ousseni Ilboudo, Joseph Ouedraogo, Elisabeth Thio, Abdoulazziz Traore, Nicolas Meda, Kouakou Kokouvi Selom Agbomadji, Richard Mawuényégan Kouamivi Agboyibor, Aléda Mawuli Badjassim, Agbégnigan Lorette Ekon, Kossi Jeff Yaka, Claver Anoumou Yaotsè Dagnra

**Affiliations:** 1 IRD, Inserm, Sciences Economiques and Sociales de la Santé and Traitement de l’Information Médicale, ISSPAM, Aix Marseille University, Marseille, 13385 France; 2 ARCAD Santé PLUS, Centre Intégré de Recherche, de Soins et d’Action Communautaire, Bamako, Mali; 3 TransVIHMI, Univ Montpellier, IRD, INSERM, Montpellier, France; 4 Espace Confiance, Abidjan, Côte d’Ivoire; 5 Association African Solidarité, Ouagadougou, Burkina Faso; 6 Espoir Vie Togo, Lomé, Togo; 7 Observatoire régional de la santé Provence-Alpes-Côte d’Azur, Marseille, France; 8 Coalition PLUS, Laboratoire de recherche communautaire, Pantin, France

**Keywords:** Men who have sex with men and women, Bisexual, Western Africa, HIV, Sexual orientation

## Abstract

HIV is highly prevalent in men who have sex with men (MSM) in West Africa. Many MSM in the region also have sex with women (MSMW). Accordingly, they are a potential bridge subpopulation for HIV transmission to women. We aimed to evaluate the proportions and characteristics of West African MSMW at high behavioral risk of acquiring HIV from male partners and transmitting it to female partners (HBRMF). The cohort ANRS-12324 CohMSM Study included 630 HIV-negative MSM in Burkina Faso, Cote d’Ivoire, Mali, and Togo. Among MSMW (i.e., with ≥ 1 female partner) in the cohort, HBRMF was identified using trajectory models based on seven at-risk sexual practices with male and female partners, including inconsistent condom use, multiple partnerships, and receptive same-sex anal intercourse. To assess the relevance of using trajectory models, we compared the proportions of participants who seroconverted during the cohort follow-up among those at HBRMF and those not at HBRMF. Factors associated with HBRMF were identified using a generalized estimation equation logistic regression model accounting for longitudinal data. Approximately half (47%) of the 304 MSMW (22% of all CohMSM study participants) were at HBRMF. This group accounted for 75% of the 28 HIV seroconversions observed during follow-up (*p* = 0.001). HBRMF was positively associated with being aged < 25 years (aOR 95% CI 1.67 [1.23–2.27]), being sexually attracted only to men (1.97 [1.38–2.78]), feelings of loneliness (1.92 [1.38–2.65]), and homonegative violence score (1.22 [1.05–1.41]). HBRMF was negatively associated with having had both stable and casual female partners in the previous 6 months (0.34 [0.20–0.60] vs. only a stable female partner). HBRMF tended to be negatively associated with having ≥ 4 sexual intercourses with female partners in the previous four weeks (0.54 [0.27–1.06] vs. no intercourse). Establishing official relationships with women might be a strategy for young and/or stigmatized MSMW to comply with social pressure to display a heterosexual lifestyle. However, this seems to increase the risk of HIV transmission to female partners. In the present study, almost half of MSMW were at HBRMF. This result stresses the need to adapt HIV research and prevention to MSMW and their female partners.

## Introduction

The HIV epidemic in West Africa is considered a “mixed epidemic.” In the general population, although prevalence is relatively low (1–2%) compared to generalized epidemics in other regions of Sub-Saharan Africa (≥ 10% in Southern Africa, 3–7% in Central and Eastern Africa), it is higher than in Northern Africa and in Western countries with concentrated epidemics (< 0.5%) (AIDSinfo | UNAIDS, 2021). In some highly stigmatized and/ or mobile groups in West Africa, HIV prevalence is very high [5–30% in men who have sex with men (MSM) and 5–20% in female sex workers (AIDSinfo | UNAIDS, 2021), 8% in gold-miners in Mali (Sagaon-Teyssier et al., 2017)]. This alarming situation prompted the international HIV prevention community to estimate to what extent these key groups act as bridge populations for HIV infection to the general population (Djomand et al., 2014; Wilson & Halperin, 2008).

It is therefore relevant to investigate the characteristics of persons who have sexual relationships both with (other) individuals from key populations and from the general population, and to identify the profiles of those at high behavioral risk of acquiring HIV and of transmitting it. In West African MSM, where HIV incidence rates can reach 16% per year (Dah et al., 2021; Dramé et al., 2013; Nowak et al., 2019), high levels of stigma and discrimination lead to poor access to health services and HIV prevention (Smith et al., 2009; Stahlman et al., 2016). Having female sexual partners was suggested to be common in West African MSM (Aho et al., 2014; Dah et al., 2016; Lahuerta et al., 2018; Larmarange et al., 2009; Mason et al., 2013; Ouedraogo et al., 2019; Papworth et al., 2014); this may be partly due to high levels of homonegativity (understood as discrimination, stigma or violence, anticipated, perceived, or experienced as a result for same-sex behaviors) (Antebi-Gruszka & Schrimshaw, 2018; Herek et al., 2005; Stahlman et al., 2017), possibly pushing MSM to engage in sexual relationships with women. These behaviors may contribute to the ongoing HIV epidemics in general populations in the region. Moreover, women account for 58% of new infections in Western and Central Africa (Joint United Nations Programme on HIV/AIDS (UNAIDS), 2020). In studies on MSM conducted in Burkina Faso, Togo, Mali, and Cote d’Ivoire, approximately half the participants declared having recent female partners, while a quarter declared multiple recent female partners. Moreover, 15–40% declared not wearing a condom during their most recent intercourse with a woman (Hakim et al., 2015, 2017; Ouedraogo et al., 2019; Ruisenor-Escudero et al., 2019). Studies in Africa investigating whether bisexual behaviors and bisexual orientation are protective factors or risk factors for at-risk sexual behaviors, HIV, and STI, have provided contrasting results (Beyrer et al., 2010; Hakim et al., 2015; Kajubi et al., 2008; Moran et al., 2020; Ouedraogo et al., 2019). Consequently, more data in West Africa are needed about the risk of MSMW HIV infection from male partners and transmission to female partners.

The present study aimed to evaluate the proportion of MSMW at high behavioral risk of acquiring HIV from male partners and transmitting it to female partners in MSM followed up in CohMSM study, a cohort study conducted in four West African countries, and to explore their various characteristics, especially dyadic characteristics regarding female partner(s).

## Method

### Participants and Procedure

The ANRS-12324 CohMSM study was a prospective cohort study implemented from June 2015 to December 2017 in Bamako (Mali), Ouagadougou (Burkina Faso), Abidjan (Côte d’Ivoire), and Lomé (Togo). It aimed to evaluate the feasibility of a comprehensive HIV prevention package for MSM in community-based clinics (Coulaud et al., 2020a, 2020b; Dah et al., 2019, 2021; Kounta et al., 2019).

Eligibility criteria were being biologically male, aged ≥ 18 years and reporting at least one anal sexual intercourse with one or more male partners in the three months prior to enrollment. Participants were offered quarterly follow-up visits in community-based clinics. These included a free clinical examination, screening, and treatment for HIV and other sexually transmitted infections (STI), individualized peer-based support, condoms and lubricants, information about risk reduction strategies, and pre- and post-HIV diagnosis counseling. Immediate antiretroviral therapy (ART) was proposed to HIV-positive participants. Using a standardized face-to-face questionnaire, trained research assistants collected demographic and socioeconomic data once at baseline (M0) and psychosocial and behavioral data every 6 months until M30. A complete description of the CohMSM study is available elsewhere (Coulaud, 2019).

Only HIV-negative MSMW (i.e., reporting ≥ 1 female partner –understood as a cisgender woman–during follow-up) who actually completed the questionnaire at baseline (M0) were included in the present analysis. Data of participants who seroconverted during the follow-up were censored at the time of HIV seroconversion.

### Measures

The outcome in this article was constructed by performing an intermediary analysis using the group-based multi-trajectory model (GBTM) (Coulaud et al., 2020b) to identify groups of MSM with similar patterns in terms of behavioral risk over time (Nagin & Odgers, 2010; Nagin & Tremblay, 2001). The analysis was performed separately for sexual behaviors with male and female partner(s) (see Table 2 for more details about the identification of trajectories).

#### Estimating Behavioral Risk Trajectories

In line with previous research on risk behaviors for HIV transmission between MSM (Gilbart et al., 2004; Hawkins, 2001; Koblin et al., 2006; Lavoie & Fisher, 2017; Patel et al., 2014), the following four sexual behaviors with male partner(s) at baseline and at each of the six-monthly follow-up surveys were introduced in the model:

1. Condom use during receptive or insertive anal sex with male partner(s) (inconsistent—“almost always,” “sometimes,” “never” used in the previous 6 months or “not used during most recent anal intercourse” vs. consistent—“always” and “used during most recent anal intercourse,” or no anal intercourse over time period)
2. Condom use during oral sex with male partner(s) (inconsistent vs. consistent as per previous definition, or no oral sex over time period)
3. Position during anal intercourse with male partner(s) (receptive or versatile vs. exclusively insertive)
4. Number of male partners (≥ 2 male partners vs. 1)

Similarly, in line with previous research on risk behaviors for heterosexual HIV transmission (Kenyon et al., 2016; Stannah et al., 2020; Weller & Davis‐Beaty, 2002), three sexual behaviors with female partner(s) at baseline and at each of the six-monthly follow-up surveys were introduced in the model:

1. Condom use during vaginal sex (inconsistent vs. consistent as per definition above, or no vaginal sex)
2. Condom use during anal sex with female partner(s) (inconsistent vs. consistent condom use as per definition above, or no anal sex)
3. Number of female partners (≥ 2 female partners vs. 1)

The optimal number of trajectories for each of the indicators for sexual behaviors with male and with female partners was identified based on the joint estimation of a polynomial regression—to define the shape of the trajectories over time- and a multinomial function—to estimate posterior probabilities of trajectory membership for each individual. For each indicator, the best model is selected on the basis of the following criteria: the Bayesian information Criterion (BIC); a mean posterior probability membership of at least 0.7; and a minimum of 5% of participants in each trajectory (Jones & Nagin, 2007; Nagin & Odgers, 2010; Nagin & Tremblay, 2001). The best-fit models showed two trajectories for each sexual behavior and allowed us to construct the following risk groups: (1) “high” versus “moderate” risk with male partner(s); (2) “high” versus “moderate” risk with female partner(s).

Fisher’s exact tests were used to compare at-risk sexual behaviors according to the behavioral risk trajectories for male and female partners, to check for consistency in their construction. In order to assess the external validity of these trajectories, the proportions of participants who HIV seroconverted during the follow-up were compared between the high risk with male partners and moderate risk with male partners groups using Fisher’s exact test. The same was done for the two ‘risk with female partners’ groups.

#### Main Outcome

MSMW belonging to both the high risk with male partners and the high risk with female partners groups were classified at high behavioral risk of acquiring HIV from male partners and transmitting it to female partners. All other combinations of groups (*e.g.*, high-moderate risk; moderate-high risk, and moderate-moderate risk) were considered less likely to be an HIV transmission “bridge” population to women, and therefore not at high behavioral risk of acquiring HIV from male partners and transmitting it to female partners. The external validity of this classification was also verified by comparing the proportions of HIV seroconverted participants (Fisher’s exact test).

### Main Analysis

Factors associated with being at high behavioral risk of acquiring HIV from male partners and transmitting it to female partners were identified using a generalized estimation equation logistic regression model which took longitudinal data into account. Variables with a *p* value below or equal to 0.2 in the univariate analyses were eligible for the multivariable analysis (backwards selection).

The following variables at baseline were considered:

*Sociodemographic and economic characteristics*: city of residence (Ouagadougou, Lomé, Abidjan, Bamako), age (< 25 y vs. ≥ 25 y), educational level (secondary school or less vs. higher than secondary school), employment status (employed vs. unemployed or manual worker vs. student), self-perceived financial situation (“difficult” or “very difficult” vs. “comfortable” vs. “just making ends meets”)

The following time-varying variables (corresponding to the 6 months prior to baseline and to each follow-up survey) were included in the analysis:

*Psychosocial characteristics*

- Sexual attraction (to men only vs. to men and women)
- Feelings of loneliness (yes vs. no), self-esteem score (assessed from 10 to 20 using the Rosenberg scale, with a score < 15 indicating low self-esteem (Rosenberg, 1965)), psychological support from friends (low vs. high), material support from friends (low vs. high)
- MSM status disclosed to at least one family member (yes vs. no), homonegative violence experience score (ranging from 4 to 16 and corresponding to the aggregate score from responses about experience of harassment or verbal aggression, physical aggression, sexual blackmail, and rape because of MSM status. For each of these four dimensions, the respondent could answer “never,” “once,” “more than once,” or “frequently” experienced homonegativity, which were scored 1, 2, 3, or 4, respectively), being a member of a local LGBT organization (yes vs. no)

*Behavioral characteristics*

- Type of female partner in the previous 6 months (stable female partner only, casual female partner(s) only, both stable and casual female partners, no female partner), relationship duration with stable female partner (< 1 year or no stable female partner in the previous 6 months vs. ≥ 1 year)
- Number of sexual intercourses with stable and/or casual female partner(s) in the previous four weeks (0 to 4 vs. ≥ 5)
- Transactional sex (i.e., receiving money from male partner(s)) in the previous 6 months

## Results

### Characteristics of Study Population

Of the 630 HIV-negative MSM enrolled in the CohMSM study cohort, the 304 MSMW were included in the present study. Socioeconomic characteristics at baseline of the 304 MSMW included in the present study are presented in Table 1. Median age was 23.9 y (interquartile range IQR 21.9–27.5). One-third were students, and one-third were unemployed or manual workers. Two-thirds reported a difficult or very difficult financial situation.

**Table 1:** Characteristics of study sample at both baseline (*n* = 304 men who have sex with men and women) and during follow-up (916 observations), and their association with high behavioral risk of acquiring HIV from male partner(s) and transmitting it to female partner(s) in univariable and multivariable analyses

	N(%) or median [IQR]	Association with high behavioral risk of acquiring HIV from male partner(s) and transmitting it to female partner(s)			
		Univariable analysis		Multivariable analysis	
		OR 95% CI	*p* value	Adjusted OR 95% CI	*p* value
*Baseline sociodemographic and economic characteristics* (*n* = 304 *participants*)					
City					
Ouagadougou	55 (18)	1.64 [1.13–2.38]	.009	1.35 [0.84–2.16]	.211
Lomé	68 (22)	1.46 [1.04–2.07]	.031	1.14 [0.74–1.79]	.544
Abidjan	61 (20)	0.77 [0.53–1.12]	.171	0.60 [0.38–0.93]	.024
Bamako	120 (39)	Ref		Ref	
Age	23.9 [21.9–27.5]				
18–24 years	141 (46)	1.56 [1.20–2.04]	.001	1.67 [1.23–2.27]	.001
≥ 25 years	163 (54)	Ref		Ref	
Educational level					
Secondary school or less	101 (33)	Ref		Ref	
Higher than secondary school	174 (57)	1.27 [0.96–1.68]	.099	1.35 [0.98–1.86]	.068
Employment status					
Employed	35 (12)	0.92 [0.60–1.41]	.693		
Unemployed or manual worker	106 (35)	0.90 [0.66–1.22]	.507		
Student	111 (36)	Ref			
Self-perceived financial situation					
Difficult/very difficult	202 (66)	Ref		Ref	
Comfortable/just making ends meet	73 (24)	1.33 [0.96–1.81]	.078	1.86 [1.29–2.67]	.001
*Psychosocial, dyadic, and behavioral characteristics during follow-up* (*n* = 916 *observations*)					
*Psychosocial characteristics*					
Sexual attraction					
To men only	329 (36)	2.24 [1.67–3.00]	.000	1.97 [1.38–2.78]	.000
To men and women	587 (64)	Ref		Ref	
Feelings of loneliness					
Yes	521 (57)	2.11 [1.57–2.83]	.000	1.92 [1.38–2.65]	.000
No	322 (35)	Ref		Ref	
Self-esteem score	14 [10–19]	0.98 [0.95–1.02]	.281		
Psychological support from friends					
High	580 (63)	0.91 [0.67–1.24]	.567		
Moderate/low/no support	263 (29)	Ref			
Material support from friends					
High	703 (77)	0.81 [0.56–1.17]	.267		
Moderate/low/no support	140 (15)	Ref			
Disclosure of MSM status to at least one family member					
Yes	321 (35)	1.46 [1.10–1.95]	.010		
No	522 (57)	Ref			
Experienced homonegative violence in the previous 6 months *Mean [min–max]*	5.5 [4–16]	1.28 [1.14–1.44]	.000	1.22 [1.05–1.41]	.009
Member of local LGBT organization					
Yes	147 (16)	Ref			
No	696 (76)	0.77 [0.53–1.12]	.171		
*Dyadic and behavioral characteristics*					
Type of female partner(s) in the previous 6 months					
Stable female partner only	434 (47)	Ref	Ref		
Casual female partner(s) only	69 (8)	0.88 [0.52–1.49]	.642	0.78 [0.44–1.36]	.375
Both stable and casual female partners	177 (19)	0.28 [0.18–0.42]	.000	0.34 [0.20–0.60]	.000
No female partner	236 (26)	2.00 [1.44–2.79]	.000	1.55 [1.07–2.25]	.019
Relationship duration with stable female partner					
< 1 year or not in a relationship with stable female partner	539 (59)	Ref			
≥ 1 year	273 (30)	0.48 [0.36–0.65]	.000		
Number of intercourses with female partner(s) in the previous 4 weeks					
0–4	804 (88)	Ref		Ref	
≥ 5	112 (12)	0.20 [0.12–0.32]	.000	0.54 [0.27–1.06]	.071
Transactional sex (received money for sex from male partner(s)) in the previous 6 months					
Yes	258 (28)	1.56 [1.15–2.10]	.004	1.38 [0.97–1.97]	.073
No	658 (72)	Ref		Ref	

IQR: Interquartile range; NI: not included in the analysis; OR: odds ratio

Table 1 also shows the psychosocial, dyadic, and behavioral characteristics corresponding to 916 observations during follow-up. Sexual attraction only to men was reported in one-third of observations, as was disclosure of MSM status to at least one family member. The median self-esteem score was low (14 IQR 10–19), and feelings of loneliness were declared in nearly two-thirds of observations. The mean score of experienced homonegative violence was 5.5 (min–max 4–16). Having a stable female partner was reported in 66% of observations, and relationship duration ≥ 1 year in 30%. Having had at least one casual female partner in the previous 6 months was reported in 27% of observations.

#### Construction of Behavioral Risk Trajectories

Tables 2, 3, and 4 show the degree of consistency of the three behavioral risk trajectories constructed according to sexual behaviors with male, female, and both male and female partners. Inconsistent condom use during anal sex and during oral sex with male partners as well as receptive or versatile position during anal sex with male partners both positively significantly contributed to being classified in the high risk with male partners group (*p* < .001), while having multiple male partners did not (*p* = .193) (Table 2). Half the participants belonged to the high risk with male partners group. There were significantly more HIV seroconversions in the ‘high risk with male partners’ group than in the moderate risk with male partners group (89% of the 28 HIV seroconversions observed, *p* < .001).

**Table 2:** Behavioral risk trajectories according to sexual behaviors with male partners and HIV seroconversions in men who have sex with men and women

	High risk with male partners *n* (%*)	Moderate risk with male partners *n* (%*)	*p* value**
Construction of behavioral risk trajectories			
Number of observations in the previous 6 months (*n* = 916)	488 (53)	428 (47)	
Sexual behaviors with male partners in the previous 6 months			
Inconsistent condom use during anal sex with male partners	243 (60)	165 (40)	.001
Inconsistent condom use during oral sex with male partners	403 (70)	173 (30)	.000
Having a receptive/versatile position during anal sex with male partners	422 (94)	26 (06)	.000
Having two or more male partners	300 (55)	245 (45)	.193
Distribution according to behavioral ‘risk with male partners’ trajectories			
Number of participants (*n* = 304)	153 (50)	151 (50)	
Number of HIV seroconversions (*n* = 28)	25 (89)	3 (11)	.000

*Percentages in line; **Proportion test (Fisher’s exact test)

**Table 3:** Behavioral risk trajectories according to sexual behaviors with female partners and HIV seroconversions in men who have sex with men and women

	High risk with female partners *n* (%*)	Moderate risk with female partners *n* (%*)	*p* value**
Construction of behavioral risk trajectories			
Number of observations in the previous 6 months (*n* = 916)	758 (83)	158 (17)	
Sexual behaviors with female partners in the previous 6 months			
Inconsistent condom use during anal sex with female partners	14 (58)	10 (42)	.004
Inconsistent condom use during vaginal sex	289 (86)	46 (14)	.032
Having two or more female partners	85 (42)	119 (59)	.000
Distribution according to behavioral ‘risk with female partners’ trajectories			
Number of participants (*n* = 304)	254 (84)	50 (16)	
Number of HIV seroconversions (*n* = 28)	23 (82)	5 (18)	.002

*Percentages in line; **Proportion test (Fisher’s exact test)

**Table 4:** Behavioral risk trajectories according to sexual behaviors with male and female partners and HIV seroconversions in men who have sex with men and women

	High risk of acquiring HIV from male partner(s) and transmitting it to female partner(s)^a^ *n* (%*)	Other MSMW^b^ *n* (%*)	*p* value**
Distribution according to behavioral ‘risk with male and female partners’ trajectories			
Number of observations in the previous 6 months (*n* = 916)	434 (47)	482 (53)	
Number of participants (*n* = 304)	137 (45)	167 (55)	
Number of HIV seroconversions (*n* = 28 participants)	21 (75)	7 (25)	.001

^a^Classified as high risk of acquiring HIV from male partner(s) and transmitting it to female partner(s) if at high risk of infection by male partners and at high risk of transmission to female partners

^b^At moderate risk of infection by male partners and/or at moderate risk of transmission to female partners

*Percentages in line; **Proportion test (Fisher’s exact test)

Inconsistent condom use during anal sex with female partners and during vaginal sex positively significantly contributed to being classified in the high risk with female partners group, while multiple female partnerships negatively contributed to being classified in it (*p* < .05) (Table 3). Eight four percent of participants belonged to the high risk with female partners group. There were significantly more HIV seroconversions in the high risk with female partners group than in the moderate risk with female partners group (82% of the 28 HIV seroconversions, *p* = .002).

Participants at high risk with both male and female partners—in other words, participants at high behavioral risk of acquiring HIV from male partners and transmitting it to female partners (i.e., the main study outcome) (Table 4)—represented 47% of observations and 45% of the study participants. The external validity of the high behavioral risk of acquiring HIV from male partners and transmitting it to female partners outcome was confirmed by the higher number of HIV seroconversions in MSMW classified at high behavioral risk of acquiring HIV from male partners and transmitting it to female partners (75% of the 28 HIV seroconversions, *p* = .001). MSMW at high behavioral risk of acquiring HIV from male partners and transmitting it to female partners accounted for 22% of the 630 MSM participants enrolled in the CohMSM study cohort.

In multivariable analysis (Table 1), high behavioral risk of acquiring HIV from male partners and transmitting it to female partners was significantly positively associated (*p* < .05) with being aged < 25 years (adjusted OR 95% confidence interval 1.67 [1.23–2.27]), self-perceiving one’s financial situation as “comfortable” or “just making ends meet” (1.86 [1.29–2.67], vs. “difficult” or “very difficult”), being sexually attracted only to men (1.97 [1.38–2.78]), feelings of loneliness (1.92 [1.38–2.65]), and homonegative violence score (1.22 [1.05–1.41]). High behavioral risk of acquiring HIV from male partners and transmitting it to female partners tended to be positively associated (0.05 < *p* < .1) with having an educational level higher than secondary school (1.35 [0.98–1.86]) and transactional sex (1.38 [0.97–1.97]). It was significantly negatively associated (*p* < .05) with having had both stable and casual female partners (0.34 [0.20–0.60], compared to a stable female partner only) in the previous 6 months. Finally, it tended to be negatively associated (0.05 < *p* < .1) with ≥ 5 intercourse with female partner(s) in the previous four weeks (0.54 [0.27–1.06]) compared to no intercourses.

## Discussion

Half of the MSM in the CohMSM study cohort were MSMW and constituted the sample in the present study. Worryingly, nearly half of these MSMW were at high behavioral risk of acquiring HIV from male partners and transmitting it to female partners, and most of the HIV seroconversions in MSMW during follow-up occurred in the high behavioral risk of acquiring HIV from male partners and transmitting it to female partners group. This group therefore represented over a fifth (22%) of the whole population of MSM followed up in CohMSM study.

### HIV Transmission Bridging Risk Between Men Who Have Sex with Men and Women and Women in West Africa

The high risk with female partners group, which accounted for four fifths of seroconversions in the study, engaged in more unprotected vaginal intercourse but multiple female partners was less frequent, compared to the moderate risk with female partners group. This implies that the risk of transmission to female partners in the high risk with female partners group is more ‘individual’ (i.e., more frequent at-risk sexual practices with female partners) rather than being dependent on the number of women exposed to the risk (i.e., fewer female partners). In addition, MSMW at high behavioral risk of acquiring HIV from male partners and transmitting it to female partners tend to have less frequent intercourse with their female partners compared to other MSMW. Although this would suggest a lower risk of transmitting HIV to women, more research is needed, especially given the high number of HIV seroconversions in MSMW at high behavioral risk of acquiring HIV from male partners and transmitting it to female partners in the present study.

The extent to which MSMW constitute an HIV transmission bridge to their female partner(s) is unclear in West Africa. In Sub-Saharan Africa, viral genotype data have produced inconsistent results regarding the overlap between the MSM and general population HIV-1 epidemics (Beyrer et al., 2012; Bezemer et al., 2014; Leye et al., 2013). The Mode of Transmission Studies estimated that MSM and their stable female partners account for 1.0–2.1% and 0.4–0.6%, respectively, of new infections in Burkina Faso, and 6.5–19% and 0.8–1.9%, respectively, of new HIV infections in Cote d’Ivoire (Gouws & Cuchi, 2012; UNAIDS, 2010).

### Psychosocial and Behavioral Context of Sexual Risk with Male and Female Partners

High behavioral risk of acquiring HIV from male partners and transmitting it to female partners was more frequent in younger MSMW, and in those with a better financial situation and a higher education level. This is consistent with several reviews showing a higher likelihood of HIV-risk behaviors and HIV infection in Sub-Saharan African young men with a high educational and socioeconomic status. The reason for this is unclear (Berhan & Berhan, 2015a, 2015b; Fortson, 2008; Hargreaves & Glynn, 2002), but could be related to greater social pressure and stigma and/or easier access to sexual partners in higher socioeconomic groups.

Our study suggests that psychosocial issues, especially those related to MSM status and sexual orientation, may be conducive to at-risk sexual behaviors with both male and female partners: Feelings of loneliness and experiencing homonegative violence were associated with high behavioral risk of acquiring HIV from male partners and transmitting it to female partners, and therefore with seroconversions. This is consistent with previous research where associations between these two variables and at-risk sexual behaviors with male partner(s) and seroconversions were shown. Research in Nigeria suggests that the effect of homonegative stigma is possibly mediated by suicidal ideation (Rodriguez-Hart et al., 2017). One study suggested that the association between feelings of loneliness and HIV-risk behavior is possibly mediated by the search for a sexual partner out of a desire for emotional/social connection. It also suggested that remorse over HIV-risk behaviors reinforced a negative self-image and feelings of loneliness (Hubach et al., 2012). Moreover, internalized homonegativity, which might result from experiencing homonegative stigma or violence, was associated with less condom use with female partners in a previous study on MSMW from the CohMSM study cohort (Fiorentino et al., 2021), and with at-risk sexual behaviors between MSM elsewhere in Sub-Saharan Africa (Ross et al., 2013). Overall, our study suggests complex interactions between HIV-risk behaviors, homonegativity, violence, and psychosocial issues, referred to as a “syndemic” of risk factors in MSM ((Mustanski et al., 2007; Stall et al., 2003).

In the present study, MSMW at high behavioral risk of acquiring HIV from male partners and transmitting it to female partners were more likely than other MSMW to have only stable (and not casual) female partners, and less likely to have frequent intercourse with female partners. High behavioral risk of acquiring HIV from male partners and transmitting it to female partners was also positively associated with being sexually attracted only to men, and being aged < 25 years. We may hypothesize that establishing official relationships with women could be a way for young and/or stigmatized MSMW to comply with social pressure to display a heterosexual way of life, while keeping sexual activity to a minimum with stable female partners, and not being particularly interested in sex with casual female partners. Engaging in relationships with women in order to hide sexual preferences for men rather than because seeking sexual pleasure or love, or hoping to “escape” from homosexuality, has been reported in other African settings where same-sex behaviors are highly stigmatized (Aho et al., 2014; Larmarange et al., 2009; Onyango-Ouma et al., 2006).

Among MSMW in CohMSM study and in other studies conducted in the same countries, recent female partners of MSM were mainly stable (Aho et al., 2014; Hakim et al., 2018). As was the case for us, the MSM populations in those studies were young, which suggests that young MSMW who are unmarried have stable relationships with women, possibly driven by the psychosocial factors described above. The fact that these partners are more stable than casual reinforces the risk of MSMW HIV bridging to women, as the present study and other studies in Togo and Burkina Faso found that condomless sex was more frequent with stable female partners than with casual female partners(Ouedraogo et al., 2019; Ruisenor-Escudero et al., 2019). MSMW may be reluctant to use condoms with their stable female partners, to avoid suspicion about extramarital activity, *a fortiori* with other men, as shown in other studies in Sub-Saharan Africa (Adedimeji et al., 2019). One hypothesis is that among older MSM, sexual risk with stable female partners might increase with marriage and with the desire to have children.

### Limitations and Strengths

The main limitation of the present study is that MSMW are still a hidden population in the four study countries. Men with secret MSM activity are more likely to (1) have female partner(s) because of social pressure, (2) avoid community-based organizations, due to fear of disclosure (O’Leary & Jones, 2006), and (3) seek other MSMW as partners, as they are considered more discrete than MSM partners in the “openly out” MSM circle where gay self-identification is stronger (Thomann et al., 2020). In line with other studies on MSM in West Africa, the present sample was young (Aho et al., 2014; Hakim et al., 2017), which further suggests that older and perhaps married MSM are more difficult to reach. Our results are therefore not representative of the full population of MSMW in Mali, Burkina Faso, Cote d’Ivoire, and Togo. However, collaborating with community-based organizations is currently the only practical way to conduct research in HIV-negative MSM in West Africa. Although we that found half of CohMSM study’s participants were MSMW, and that a fifth were at high behavioral risk of acquiring HIV from male partners and transmitting it to female partners, the potential for MSMW to act as a bridge population for HIV transmission to women may nonetheless have been underestimated.

A second study limitation is possible social desirability bias, which may have led some MSMW in our study to over-declare the number of female partners or to under-declare at-risk sexual behaviors. However, one of the strengths of the study was that it used longitudinal data on psychosocial and behavioral characteristics and also on HIV status. Accordingly, the accuracy of self-reported behaviors used to build our behavioral risk trajectories was validated by the higher number of HIV seroconversions which occurred in all three high risk groups over the 18-month follow-up period. We did not have data on HIV seroconversions of female partners of these MSMW, but these would only have strengthened our findings.

Another strength of our study was to combine at-risk sexual behaviors with both male and female partners (inconsistent condom use during anal sex with male and with female partner(s), during vaginal sex, and position type during anal sex with male partners), into a multifactorial outcome to identify the behavioral trajectories of MSMW at greatest risk of transmitting HIV to women. In previous studies on MSM in West Africa, some of these variables, analyzed separately, were not associated with HIV-positive status (Ouedraogo et al., 2019; Ruisenor-Escudero et al., 2019).

### Implications for HIV Research and Prevention

The results of this study highlight the need for MSMW to increase and adapt HIV prevention in their female sexual partnerships. Accordingly, it is critical to (1) reinforce community-based organizations and advocate better social acceptance of MSM in order to better reach hidden MSM and (2) investigate male bisexuality in West Africa through psychosocial, behavioral, phylogenetic, and epidemiological research on MSMW and on their female partners.

Qualitative research on West African MSM has shown very diverse reasons to have female partners, including a desire to hide one’s MSM status, a desire to have children, sexual attraction, love, and the need to have a confidant (Enel et al., 2009; Larmarange et al., 2009). More research on behaviors with spouses and girlfriends (aware or not of their partner’s MSMW status), casual female partners, and female sex workers would inform choices on how, if possible, to adapt HIV prevention to various dyadic situations. In men who sell sex to other men to cope with a difficult financial situation, the desire to have female partners—and therefore their behaviors with them—might differ from those of other MSMW (Bui et al., 2014). Although the present study focused on the behavioral risk of HIV transmission by MSMW to women, the risk of HIV transmission from women to MSM also needs to be explored. In particular, subpopulations of female partner(s) of MSM (e.g., female sex workers and female clients of MSM sex workers) may present specific HIV behavioral risk dynamics.

The self-perceived risk to acquire HIV from male partner(s) and therefore to transmit it to female partner(s) might be underestimated by MSMW. In previous MSM studies in Cote d’Ivoire and Burkina Faso, half of the participants thought that the risk of HIV transmission was higher or similar in vaginal intercourse than in anal intercourse, and higher or similar during intercourse with women than with men (Dah et al., 2016; Hakim et al., 2015; Moran et al., 2020; Ouedraogo et al., 2019). Raising awareness in MSMW about the risk of HIV transmission to their stable female partners, and indirectly to their children through mother-to-child transmission, might help them to improve their HIV prevention strategies with male partners. Another important barrier to HIV prevention in MSMW might be non-disclosure of extramarital relationships to their stable female partners, *a fortiori* relationships with men, which concerns most MSMW (Ekouevi et al., 2014; Lahuerta et al., 2018) and jeopardizes condom negotiation. Encouraging MSMW to use condoms with a stable female partner might be difficult as they may feel they need to comply with heteronormative norms and to have children (Adedimeji et al., 2019; Broqua, 2010; Enel et al., 2009). Consequently, reinforcing knowledge and access to pre-exposure prophylaxis (PrEP) in MSMW is key to preventing HIV transmission from MSMW to women. However, specific facilitators and barriers to PrEP-use in this context may exist and should be explored. Similarly, investigating facilitators and barriers to HIV testing, linkage to care, and antiretroviral therapy adherence in HIV-positive MSMW would help to improve the HIV cascade care and reduce the HIV bridging risk to women.

Overall, the present study—reflecting previous results from the CohMSM study cohort (Fiorentino et al., 2021)—suggests that homonegativity not only reinforces MSM psychosocial and health vulnerabilities, but also leads to public health issues regarding the risk of MSMW transmitting HIV to women. This highlights that advocacy against existing homonegativity not only concerns human rights but public health too.

## Data Availability

Due to French law, there are restrictions on publicly sharing the data of this study. French law requires that everyone who wishes to access cohorts data or clinical study data on humans must ask the French data protection authority, la Commission Nationale de l'Informatique et des Libertés (CNIL), for permission by filling a form which can be provided by Bruno Spire (mail: bruno.spire@inserm.fr). For further information, please see: https://www.cnil.fr/.
